# Molecular Control of Flower Colour Change in Angiosperms

**DOI:** 10.3390/plants14142185

**Published:** 2025-07-15

**Authors:** Fernanda M. Rezende, Magdalena Rossi, Cláudia M. Furlan

**Affiliations:** Department of Botany, Institute of Biosciences, University of São Paulo, São Paulo 05508-090, SP, Brazil; mmrossi@usp.br (M.R.); furlancm@ib.usp.br (C.M.F.)

**Keywords:** anthocyanins, betalains, carotenoids, plant pigment, transcription factor, transcriptional regulation, post-transcriptional regulation, epigenetics

## Abstract

Floral pigmentation contributes directly to reproductive strategies and fitness by shaping pollinator behaviour, and its regulation therefore represents a critical aspect of flower development. Additionally, it is a major determinant of aesthetic and economic value in the ornamental plant industry. This review explores the genetic, biochemical, and ecological bases of floral colour change, focusing on the biosynthesis and regulation of the three major classes of plant pigments: carotenoids, flavonoids (particularly anthocyanins), and betalains. These pigments, derived from primary metabolism through distinct biosynthetic pathways, define the spatial and temporal variability of floral colouration. We discuss the molecular mechanisms underlying flower colour change from opening to senescence, highlighting pigment biosynthesis and degradation, pH shifts, metal complexation, and co-pigmentation. Additionally, we address the regulatory networks, including transcription factors (MYB, bHLH, and WDR) and post-transcriptional control, that influence pigment production. Finally, we provide a comprehensive survey of angiosperm species exhibiting dynamic petal colour changes, emphasizing how these mechanisms are regulated.

## 1. Introduction

Flower colours are important in the interaction between plants and their pollinators. Since Darwin’s evolutionary theory, biologists have studied how floral variation influences plant reproductive success and pollination dynamics. Flower colours define pollination syndromes, which are associated with pollinator groups including bees, butterflies, birds, beetles, and others [1,2,3]. The coevolution of pollinators and angiosperms drives the floral diversity (e.g., flower morphology, colour, scent, nectar quantity, and/or quality) [4], and this resulted in reproductive isolation and plant speciation [5].

Interestingly, pollinators perceive colour through distinct visual systems, and the accumulation of specific metabolites in floral tissues can influence their preferences, even when such differences are not apparent to the human eye [6]. For example, bee-pollinated flowers are often white, purple, blue, or yellow but rarely red, which may appear black to bees [3].

Beyond ecological relevance, floral pigmentation is a major aesthetic trait in the ornamental plant industry, directly affecting the commercial value of flowers [7]. Gregor Mendel, who established the fundamental laws of genetics, was interested in plant breeding and succeeded in obtaining a new *Fuchsia* variety (Onagraceae, Myrtales) with an unprecedented flower colour [8]. Today, advances in genetic engineering have enabled the development of novel and enhanced flower colour patterns (i.e., more lasting or vibrant, or completely different colours from those of the wild genotype). A notable example is *Dianthus* (Caryophyllaceae, Caryophyllales), the first genetically modified commercial flower species, which displays a wide variation in colours ranging from white to red [9]. Historically, several plant species have served as key models in the study of flower colour. *Petunia hybrida* (Solanaceae, Solanales), for instance, has been a model for the genetic regulation of anthocyanins [10]. *Ipomoea nil* (Convolvulaceae, Solanales) has revealed mechanisms of pH-mediated colour change [11], while *Antirrhinum majus* (Plantaginaceae, Lamiales) laid the foundations of classical floral genetics [12]. In cultivated roses (Rosaceae, Rosales), efforts to engineer novel hues have led to the biosynthesis of delphinidin-based blue pigments [13].

**Figure 1:**
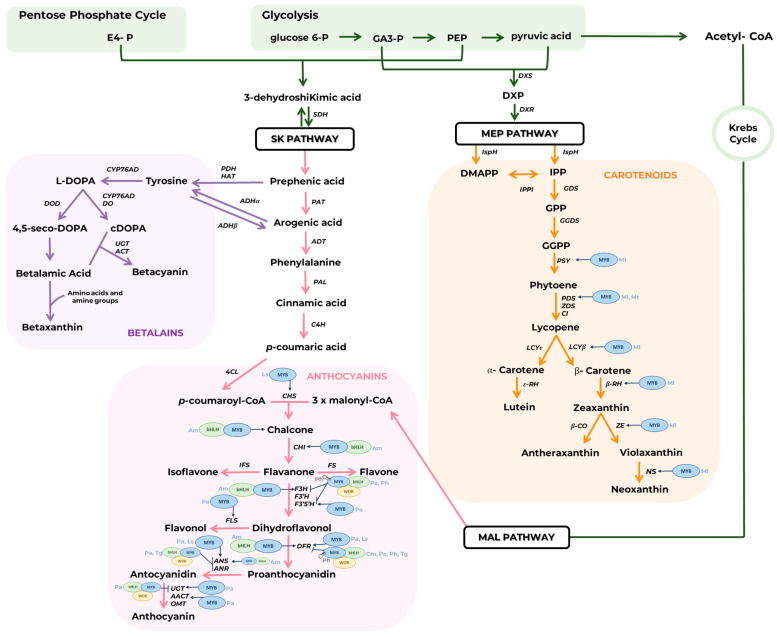
Simplified schematic representation of plant pigment biosynthetic pathways. Primary metabolism, carotenoid, flavonoid and betalain pathways are highlighted in green, orange, pink, and purple, respectively. Shikimate (SK), methylerythritol phosphate (MEP), and malonate (MAL) pathways are highlighted in boxes. The transcription factors included in the figure were described as regulators of flower pigment biosynthesis. Abbreviations indicate the following metabolites and enzymes: 1-deoxy-D-xylulose-5-P (DXP), 4-CUMAROIL-COA LIGASE (4CL), 4-HYDROXY-3-METHYLBUT-2-ENYL DIPHOSPHATE REDUCTASE (IspH), ANTHOCYANIDIN ACYL TRANSFERASE (AACT), ACYL TRANSFERASE (ACT), ANTHOCYANIDIN REDUCTASE (ANR), ANTHOCYANIDIN SYNTHASE (ANS), AROGENATE DEHYDRATASE (ADT), AROGENATE DEHYDROGENASE (ADH), CAROTENE ISOMERASE (CI), CHALCONE SYNTHASE (CHS), CHALCONE ISOMERASE (CHI), CINNAMATE 4-HYDROXYLASE (C4H), Coenzyme A (CoA), CYTOCHROME P450-TYPE GENE (CYP76AD), cyclo-DOPA (cDOPA), DIHYDROFLAVANOL 4-REDUCTASE (DFR), dihydroxyphenylalanine (L-DOPA), dimethylallyl diphosphate (DMAPP), DIPHENOL OXIDASE (DO), DOPA DIOXYGENASE (DOD), DXP REDUCTOISOMERASE (DXR), DXP SYNTHASE (DXS), erythrose 4-phosphate (E4P), FLAVANONE 3′5′-HYDROXYLASE (F3′5′H), FLAVANONE 3′-HYDROXYLASE (F3′H), FLAVANONE 3-HYDROXYLASE (F3H), FLAVONE SYNTHASE (FS), FLAVONOL SYNTHASE (FLS), geranyl diphosphate (GPP), geranylgeranyl diphosphate (GGPP), GERANYLGERANYL DIPHOSPHATE SYNTHASE (GGDS), glyceraldehyde 3-phosphate (GA3P), GPP SYNTHASE (GDS), ISOFLAVONE SYNTHASE (IFS), ISOPENTENYL DIPHOSPHATE ISOMERASE (IPPI), isopentenyl diphosphate (IPP), LYCOPENE β-CYCLASE (LCYβ), LYCOPENE ε-CYCLASE (LCYε), NEOXANTHIN SYNTHASE (NS), *O*-METHYLTRANSFERASE (OMT), PHENYLALANINE AMMONIA LYASE (PAL), PHENYLPYRUVATE AMINOTRANSFERASE (HAT), phosphoenolpyruvate (PEP), PHYTOENE DESATURASE (PDS), PHYTOENE SYNTHASE (PSY), PREPHENATE AMINOTRANSFERASE (PAT), PREPHENATE DEHYDROGENASE (PDH), SHIKIMATE DEHYDROGENASE (SDH), UDP-GLUCOSYLTRANSFERASE (UGT), ZETA-CAROTENE DESATURASE (ZDS), ZEAXANTHIN EPOXIDASE (ZE), Β-CAROTENE OXYGENASE (β-CO), β-RING HYDROXYLASE (β-RH), ε-RING HYDROXYLASE (ε-RH). Regulatory mechanisms are indicated only for flower tissues and species abbreviations are shown in light blue. Am: *Antirrhinum majus*; Cm: *Chrysanthemum morifolium*; Ls: *Lilium* sp.; Ml: *Mimulus lewisii*; Mt: *Medicago truncatula*; Pa: *Petunia axilaris*; Ph: *Petunia hybrida*; and Tg: *Tulipa gesneriana*. MYB: MYELOBLASTOSIS transcription factor. bHLH: BASIC HELIX-LOOP-HELIX transcription factor. WDR: WD40 repeat domain transcription factor. Hairpins indicate microRNAs that regulate the corresponding transcription factor.

Among floral traits, colour is one of the best-studied in terms of its genetic, biochemical, and ecological aspects. Regardless of chlorophylls, there are three classes of pigments present in vegetal tissues that confer colour to flowers: carotenoids, betalains, and flavonoids (especially anthocyanins). These specialized metabolites originated from the products of the primary metabolism by the plastidial shikimate (SK), methylerythritol phosphate (MEP), and cytosolic malonate (MAL) pathways. Carotenoids are synthesized via MEP, betalains from SK, and flavonoids arise from intermediates of SK and MAL pathways (Figure 1).

In this review, we synthesize the current knowledge on the biosynthetic pathways and regulatory networks underlying the three major classes of floral pigments. We also present an updated and expanded compilation of angiosperm species that exhibit flower colour change from opening to senescence. This expanded dataset includes taxonomic, geographic, and mechanistic information, offering a more comprehensive picture of the temporal dynamics of floral pigmentation.

## 2. Anthocyanins

Among angiosperms, flavonoids, especially anthocyanins, are the predominant floral pigments [14,15]. They are responsible for the red/orange to violet/blue colouration of fruits and flowers according to the vacuole pH [13]. Anthocyanins act as photoprotectants due to their UV absorption spectra and high antioxidant activity. In general, in non-reproductive tissues, anthocyanins accumulate in response to biotic and abiotic stresses, such as pathogens, high/low temperatures, high levels of light, UV-B radiation, drought, nutrient deficiency, high ozone concentration, etc. [16,17,18,19,20,21].

Anthocyanins are synthesized by the combination of precursors from the SK and MAL pathways, being the terminal branch of the highly conserved flavonoid pathway [22]. Flavonoids constitute a relatively diverse family of aromatic molecules synthesized in the cytosol from the plastidial precursor *L*-phenylalanine [14,23]. The first reaction in flavonoids biosynthesis is catalyzed by the CHALCONE SYNTHASE (CHS) that condensates *p*-coumaroyl-CoA with three molecules of malonyl-CoA resulting in the formation of a tetrahydroxy alcohol, which cyclizes producing chalcone. This intermediate serves as the precursor for the synthesis of flavanones (e.g., narigenin), isoflavones (e.g., genistein), flavones (e.g., apigenin), dihydroflavonols (e.g., dihydrokaempferol), flavonols (e.g., kaempferol), proanthocyanidins (e.g., epicatechin polymers), anthocyanidins (e.g., cyanidin), and anthocyanins (e.g., cyanidin-3-*O*-glucoside) [24,25] (Figure 1). Dihydroflavonol is the branching point for the synthesis of flavonol by FLAVONOL SYNTHASE (FLS) and proanthocyanidin by DIHYDROFLAVONOL 4-REDUCTASE (DFR). Anthocyanidin synthesis further requires the activity of ANTHOCYANIDIN SYNTHASE (ANS). Finally, depending on their substituents profile, anthocyanins are synthetized by the action of UDP GLYCOSYL TRANSFERASE (UGT), ANTHOCYANIDIN ACYL TRANSFERASE (AACT), and/or O-METHYL TRANSFERASE (OMT). Anthocyanidins are the aglycones and chromophores of anthocyanins (glycosylated compounds), probably the best-known and most studied group of flavonoids [24,26,27,28].

Out of more than twenty described anthocyanidins, three are particularly abundant: cyanidin (brick red/magenta), delphinidin (blue/purple), and pelargonidin (orange/red). In nature, anthocyanins typically accumulate in the vacuoles and provide a wide range of colours. The difference in colour tones is determined by the substitution patterns in the core structure of the B-ring (e.g., hydroxyl and methoxy) and in the A- and C-ring (e.g., glycosylations and acylations) [29,30].

Additionally, the colour also depends on the vacuolar pH and the presence of copigments. At very acidic pH levels (<2.5), they predominantly appear as the coloured flavylium cation. Around pH 3, however, most anthocyanins undergo nucleophilic attack by water, forming a colourless or pale-yellow hemiketal structure. This form can further rearrange into *cis*- or *trans*-hydroxychalcones, which are also colourless or pale-yellow [31]. Additionally, anthocyanins containing two or more adjacent hydroxyl groups in the B-ring can form complexes with metal ions, such as Al^3+^, Fe^2+^/Fe^3+^, and Mg^2+^, leading to prominent colour changes [11,32,33]. Moreover, copigmentation can be defined as the formation of noncovalent complexes involving an anthocyanin and a copigment. The major natural copigments—hydrolysable tannins, flavonoids, and phenolic acids—modulate flower colour hue by interacting with anthocyanins through their π-conjugated systems. These interactions can stabilize anthocyanin structures, enhance colour intensity, and shift hues depending on pH, concentration, and the specific molecular environment within the vacuole. Some non-phenolic copigments have also been described, including alkaloids, amino acids, organic acids, nucleotides, and polysaccharides, but their efficiency is usually lower than polyphenols. It remains unclear whether these compounds can significantly contribute to colour expression provided by anthocyanins [30].

Anthocyanin biosynthesis is intricately regulated by a complex network of transcription factors (TFs), which includes MYELOBLASTOSIS (MYB), BASIC HELIX-LOOP-HELIX (bHLH), and WD40 repeat domain (WDR) protein families [34]. MYBs, bHLHs, and WDRs usually form a protein complex known as MBW that binds to specific motifs on the promoter regions of the structural genes modulating their expression [35,36,37].

In monocotyledons, such as maize (*Zea mays*, Poaceae, Poales) [38] and rice (*Oryza sativa*, Poaceae, Poales) [39], MYB TFs regulate the expression of key anthocyanin biosynthetic enzymes encoding genes including *CHS*, *FLAVANONE 3-HYDROXYLASE* (*F3H*), *DFR*, and *UGT* [40]. In the eudicots species, *Arabidopsis thaliana* (Brassicaceae, Brassicales) [41] and *Malus domestica* (Rosaceae, Rosales) [42], the early genes of anthocyanins biosynthesis (i.e., *CHS*, *CHALCONE ISOMERASE* (*CHI*), *F3H*, and *F3′H*) are directly regulated by MYB TFs [43]. On the contrary, the expression of late biosynthetic genes (i.e., *DFR*, *ANR*, and *UGT*) is controlled by the MBW protein complex [44,45,46].

In *Petunia axilaris* (Solanaceae, Solanales), a genome-wide analysis identified five R2R3-MYB TFs that were involved in the regulation of flower pigmentation [47]. PaMYB33/PhAN2 and PaMYB77/PaMYB78/PhAN4 play a central role in petal limb pigmentation controlling the expression of DFR and F3′5′H, respectively [47,48,49]. Other regulators include PaMYB100, responsible for vein-associated pigmentation, and PaMYB81, which mediates light-induced anthocyanin accumulation in petal surfaces. The overexpression of any of these two genes resulted in the upregulation of the general phenylpropanoid biosynthetic pathway except for *FLS*, *UGT*, and *ACT* [50]. Finally, PaMYB105 as part of the MBW complex acts as a negative regulator of anthocyanin biosynthetic genes (i.e., *F3H*, *F3′5′H*, *DFR*, *ANS*, and *UGT*) [51]. This comprehensive characterization of MYB genes in *P. axilaris* highlights both activators and repressors shaping the complexity of anthocyanin regulation (Figure 1), further illustrating the conserved yet diversified regulatory networks across eudicots.

Regulatory mechanisms involving MYB transcription factors have been described in a variety of flowering plants. For example, in *Tulipa gesneriana* (Liliaceae, Liliales), MYB4 TF was demonstrated to inhibit the *ANS* and *DFR* genes by blocking the MBW activation [52]. MYB5 was shown to positively regulate *CHS*, *DFR*, and *ANS* in lily flowers (*Lilium* sp., Liliaceae, Liliales), while MYB1 downregulates anthocyanin synthesis [53]. In the different flower developmental stages of a peony (*Paeonia suffruticosa*, Paeoniaceae, Saxifragales), MYB58 had similar transcription levels to those anthocyanin biosynthetic genes, such as *CHS*, *CHI*, *DFR*, and *ANS* [54]. Lastly, in *Antirrhinum majus* (Plantaginaceae, Lamiales) the MYB transcription factor Ros1 and the bHLH protein Delila activate structural genes, such as *CHS*, *CHI*, *F3H*, *DFR*, and *ANS*. Unlike the classical MBW complex, their activity is not enhanced by the WD40 protein (WDR1) and may even be inhibited by it. However, when Ros1 interacts with a different bHLH, Inc I, the presence of WDR1 enhances transcriptional activation, suggesting that distinct combinations of MYB, bHLH, and WDR proteins modulate anthocyanin gene expression in a context-dependent manner [55,56] (Figure 1).

Although the role of RNA interference (RNAi) in regulating the flavonoid pathway was not extensively explored in early studies, research in this area has substantially increased over the past two decades, revealing key post-transcriptional mechanisms that complement transcriptional regulation. In *Arabidopsis thaliana*, MYB TFs are targeted by miRNAs and tasiRNAs [57,58]. In kiwifruit (*Actinidia arguta*, Actinidiaceae, Ericales), miR858 acts as a negative regulator of anthocyanin biosynthesis by repressing the R2R3-MYB AaMYBC1, thereby reducing anthocyanin accumulation and pigmentation [59]. Also, in the flower tissues of *Petunia hybrida*, miR319 promotes venation patterning in the dorsal tube by repressing the R2R3-MYB (AN4) [60].

Interestingly, the external application of dsRNAs in *A. thaliana* and *Solanum lycopersicum* (Solanaceae, Solanales) has emerged as a tool for regulating endogenous genes without the need for stable transformation. Moreover, dsRNAs can be used to efficiently silence key flavonoid biosynthetic genes, including *CHS*, offering a promising non-transgenic strategy for modulating pigment production in plants [61,62].

Recent discoveries in *A. thaliana* have added further complexity to the regulatory network of anthocyanin biosynthesis. The sugar-sensing kinase SnRK1 represses anthocyanins accumulation both transcriptionally and post-translationally. SnRK1 is a negative regulator of sucrose-induced anthocyanin biosynthesis by the transcriptional repression of MYB75, an inductor of *DFR* expression. Additionally, under metabolic stress conditions, SnRK1 phosphorylates the MBW complex (MYB75/bHLH2/TTG1) leading to its dissociation, as well as the degradation of MYB75 and nuclear export of TTG1, thereby limiting pigment synthesis as a strategy to save energy [63].

Epigenetic regulation brings another layer of control [64]. In maize, chromatin remodelling has been implicated in the modulation of anthocyanin biosynthetic gene expression [65]. Additionally, the DNA methylation of regulatory regions can modulate pigment production, as demonstrated in *Xanthoceras sorbifolium* (Sapindaceae, Sapindales), a species that exhibits dynamic flower colour transitions [66].

Although anthocyanin biosynthesis is regulated by a complex network of structural and regulatory genes, recent advances in CRISPR/Cas9 technology have facilitated target gene editing, making it a powerful tool for the genetic engineering of ornamental plants [67]. In *Petunia hybrida*, the knockout of *DFR*, *F3′H*, and *F3′5′H* resulted in predictable shifts in flower colour by altering anthocyanin composition [68]. Similarly, in *Ipomoea nil*, targeted mutagenesis of the *DFR* gene successfully disrupted anthocyanin biosynthesis, resulting in white flowers [69]. In *Torenia fournieri* (Scrophulariaceae, Lamiales), *MYB1* knockout suppressed anthocyanin accumulation also resulting in white-flowered lines [70]. In *Nicotiana tabacum* (Solanaceae, Solanales), the knockout of MYB4 led to a significant reduction in anthocyanin accumulation and lighter floral colouration [71]. These examples illustrate the power of CRISPR-mediated editing to develop biotechnological strategies for flower colour engineering.

Collectively, these findings reveal a sophisticated regulatory mechanism for flavonoid biosynthesis, integrating transcriptional networks, small RNA-based regulation, post-translational modifications, and epigenetic control. This complexity ensures that anthocyanin production is finely tuned in response to developmental cues and environmental stresses, balancing pigment synthesis with energy demands and survival strategies.

## 3. Carotenoids

Carotenoids, also known as tetraterpenoids (C_40_), belong to the terpenoid class of specialized metabolites. These lipid-soluble compounds are synthesized by all photosynthetic organisms—including plants, algae, and bacteria—and some non-photosynthetic bacteria and fungi [72,73]. In photosynthetic tissues, carotenoids act as components of the light-harvesting machinery and serve as photoprotective agents, safeguarding cells from oxidative damage and being essential players for photosynthetic performance and plant survival [74,75,76]. In non-photosynthetic organs such as flowers and fruits, carotenoids accumulate in chromoplasts—most commonly formed via the transition from chloroplasts—and function as attractants for pollinators and seed dispersers [75,77,78].

Since the 19th century, carotenoids, together with other natural pigments, attracted the attention of organic chemists. β-carotene was first isolated in 1817, and by 1937, xanthophylls were identified as the pigments responsible for autumnal leaf colour [79]. Furthermore, carotenoids are vital in human nutrition, acting as precursors for vitamin A biosynthesis and functioning as antioxidants that help to prevent age-related eye degeneration, cancer, and cardiovascular diseases [80].

Yellow and orange hues in flowers and fruits are generally attributed to carotenoid accumulation [81]. For example, capsanthin and capsorubin confer the characteristic red colour of *Capsicum annuum* (Solanaceae, Solanaceae) fruits, while lutein and zeaxanthin are responsible for yellow to orange colouration in *Tagetes erecta* (Asteraceae, Asterales) [82,83]. In *Crocus sativus* (Iridaceae, Asparagales), the carotenoid crocetin imparts a deep golden hue to the stigmas harvested as saffron. These pigments not only attract pollinators and seed dispersers but also contribute to human health through their antioxidant properties and role as vitamin A precursors [84]. In some cases, carotenoids coexist with anthocyanins, producing bronze or brownish tones, and they may also contribute partially to red colouration in specific taxa [85].

The building blocks of all terpenoids are five-carbon precursor isopentenyl diphosphate (IPP) and its isomer dimethylallyl diphosphate (DMAPP), being synthetized by the cytosolic mevalonate (MEV) and the plastidial MEP pathways, respectively [86,87]. Although compartmentalized, crosstalk and metabolite exchange between the MEV and MEP pathways are well documented [88].

The MEV pathway supplies precursors for cytosolic terpenoids such as sesquiterpenes (C_15_), triterpenes (C_30_), sterols, and dolichols. The MEP pathway is responsible for the synthesis of C_5_ isoprene, C_10_ monoterpenes, C_20_ diterpenes, and C_40_ carotenoids [89,90]. The MEP pathway (Figure 1), in plastids, begins with the condensation of glyceraldehyde 3-phosphate (GA3P) and pyruvic acid via 1-DEOXY-D-XYLULOSE SYNTHASE (DXS), forming 1-deoxy-D-xylulose-5-phosphate (DXP). Subsequently, intramolecular rearrangement and a reduction in DXP by DXP REDUCTOISOMERASE (DXR) leads to MEP formation. After several steps, IPP and DMAPP are produced and undergo sequential condensation to yield geranylgeranyl diphosphate (GGPP), the immediate precursor for carotenoids. The tail-to-tail condensation of two GGPPs mediated by the activity of PHYTOENE SYNTHASE (PSY) results in phytoene, which is desaturated and isomerized into lycopene by enzymes including PHYTOENE DESATURASE (PDS), ZETA-CAROTENE DESATURASE (ZDS), and CAROTENE ISOMERASE (CI). Lycopene is a key branch point in the carotenoid pathway, undergoing cyclization by LYCOPENE CYCLASES (LCYε, LCYβ) to produce α-carotene or β-carotene. These two compounds are precursors to xanthophylls (oxygenated carotenoids), such as lutein, violaxanthin, and zeaxanthin, formed via hydroxylation catalyzed by β-RING HYDROXYLASE (β-RH) and ε-RING HYDROXYLASE (ε-RH) [85].

Genes encoding the enzymes of the carotenoid biosynthetic pathway have been identified in numerous species, and their expression is regulated by both developmental and environmental signals [81]. Some steps are light-dependent and modulated by chloroplast redox status [91]. In model species like *Solanum lycopersicum*, *Capsicum annuum* (Solanaceae, Solanaceae) fruits, and *Arabidopsis thaliana*, several transcription factors have been implicated in the transcriptional regulation of carotenoid biosynthetic genes. Among them, PHYTOCHROME INTERACTING FACTORS (PIFs, e.g., AtPIF1 and SlPIF1a) act as negative regulators under low-light conditions, while the RIPENING INHIBITOR (SlRIN), a MADS-box transcription factor, positively regulates key genes, such as PSY1 during fruit ripening [83,92,93].

Beyond bHLH and MADS-box factors, additional transcription factor families have emerged as important regulators. For example, NAC transcription factors, such as FcrNAC22, mediate red-light-induced carotenoid biosynthesis in kumquat fruits [94]. WRKY factors, like WRKY, negatively regulate carotenoid accumulation in citrus products by binding to the promoters of biosynthetic genes such as LCYB1 [95]. Similarly, MYB transcription factors, including WHITE PETAL1 (WP1) in *Medicago truncatula* (Fabaceae, Fabales) and RCP1 in *Mimulus lewisii* (Phrymaceae, Lamiales), act as positive regulators of carotenoid pigmentation in flowers, linking transcriptional control to floral development and colouration [96,97]. Thus, the transcriptional regulation of structural carotenoid biosynthetic genes—such as *PSY*, *PDS*, *LCYβ*, and *β-RH*—is highly complex and tightly integrated, leading to remarkable diversity in carotenoid composition and accumulation across tissues and species [98].

It is worth noting that red flowers derived predominantly from carotenoids are relatively rare, and their regulatory mechanisms remain less studied compared to anthocyanin-based red pigmentation [99].

Recent evidence in *Arabidopsis thaliana* highlights that post-translational mechanisms are also important in regulating carotenoid biosynthesis. Protein stability and enzyme activity are influenced by ubiquitination, phosphorylation, and interaction with molecular chaperones such as chaperone Hsp70. For instance, the key enzyme PSY is regulated through interaction with ORANGE (OR) chaperone, which stabilizes the enzyme, and is targeted for degradation via proteases or ligases [98,100,101]. Additionally, light-mediated signalling may also influence enzyme phosphorylation states, adding further complexity to carotenoid metabolic control.

Building upon this molecular understanding, recent applications of CRISPR/Cas9 technology have demonstrated its potential for directly manipulating carotenoid biosynthesis and modifying flower colouration in some species. For example, in *Ipomoea nil* (Convolvulaceae, Solanales), a targeted knockout of the *CAROTENOID CLEAVAGE DIOXYGENASE* (*CCD*) gene significantly reduced carotenoid degradation, leading to a striking shift from white to pale yellow petals and a 20-fold increase in carotenoid content, revealing the role of carotenoid cleavage in maintaining pigment levels [69]. In *Brassica napus* (Brassicaceae, Brassicales), the simultaneous CRISPR-mediated editing of two copies of *CI* triggered creamy white petals and yellowish leaves. This phenotype was associated with the transcriptional downregulation of *PSY* and *C4H* and the upregulation of *PDS*, *ZE*, *β-RH*—genes involved in both carotenoid and flavonoid biosynthetic pathways. These coordinated changes underscore the intricate regulatory networks governing pigment accumulation and demonstrate how CRISPR/Cas9 can be used to unravel and rewire pigment biosynthesis at the genetic level [102].

## 4. Betalains

**Figure 2:**
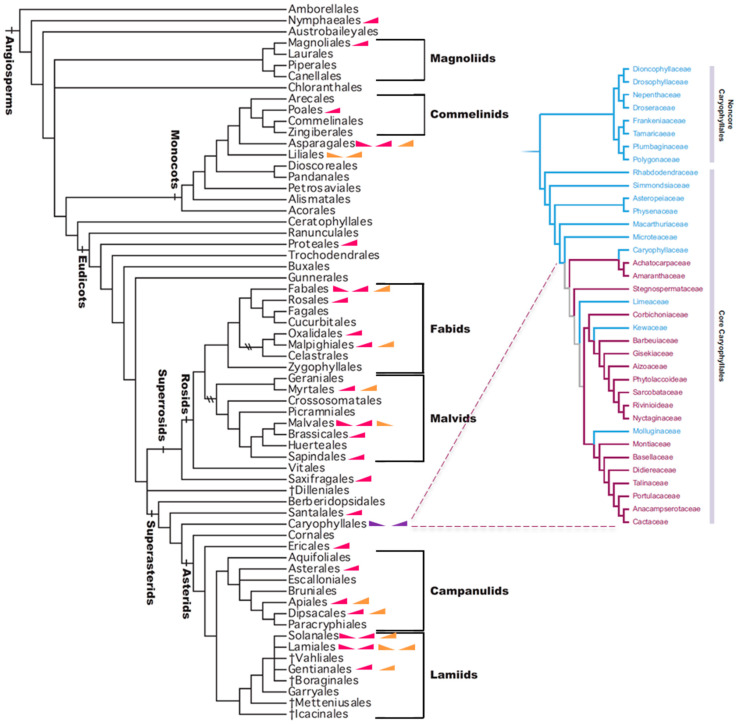
Schematic phylogeny of angiosperms showing the distribution of flower colour change from opening to senescence across major clades according to APG IV classification. Crosses (†) indicate informally recognized clades or taxa with uncertain or unresolved phylogenetic placement. Pink, orange, and purple ramps indicate anthocyanins, carotenoids, and betalains, respectively. Upward ramps indicate pigment accumulation, and downward ramps denote pigment degradation. The inset on the right highlights the phylogenetic placement of core Caryophyllales families (adapted from [103]). The clades containing betalains and anthocyanins are indicated in purple and blue, respectively.

Betalains are nitrogen-containing pigments that produce vivid red to violet (betacyanins) or yellow to orange (betaxanthins) colours and occur exclusively in certain Caryophyllales lineages, colouring vegetative and reproductive organs. This well-defined clade comprises 38 families, with 17 of those being betalain accumulators [103] (Figure 2). Betalains are considered chemosystematic markers of the core Caryophyllales [104], in which approximately 75 distinct compounds have been identified [105]. However, about one third of the species within the core Caryophyllales are known to produce anthocyanin, similar to non-core Caryophyllales lineages [103].

Betalains are derived from the amino acid *L*-tyrosine, an aromatic amino acid synthetized by the SK plastidial pathway (Figure 1). The first step of betalain biosynthesis involves the hydroxylation of tyrosine to form dihydroxyphenylalanine (*L*-DOPA). Recent studies have shown that in plants, this reaction is catalyzed by members of the cytochrome P450 enzyme family, particularly CYP76AD and its homologs, which also mediate the formation of cyclo-DOPA (cDOPA). These findings reveal a broader enzymatic flexibility at the initial steps of the betalain pathway, highlighting the dual functionality of CYP76AD enzymes in initiating pigment biosynthesis [103,106,107]. *L*-DOPA is an important precursor not only of betalains but also of other specialized metabolites in plants [81]. The cleavage of *L*-DOPA mediated by DOPA 4,5-DIOXYGENASE (DOD) followed by non-enzymatical rearrangements resulting from the formation of betalamic acid, the core structure of betalains [105,107]. Betalamic acid is the chromophore molecule of both betacyanins (e.g., betanin) and betaxanthins (e.g., vulgaraxanthin), which impart violet and yellow colouration, respectively [81]. Its condensation with cDOPA results in the formation of betacyanins, while the condensation with amino acids or amine groups leads to the formation of betaxanthins [105,107] (Figure 1).

Recent evolutionary studies with leaves of *Beta vulgaris* (Amaranthaceae, Caryophyllales) revealed that the Caryophyllales species producing betalains possess a duplicated AROGENATE DEHYDROGENASE (ADHα) gene variant that has lost sensitivity to feedback inhibition by tyrosine. This metabolic innovation ensures sustained *L*-DOPA production even under high tyrosine levels, facilitating the high-pigment-accumulation characteristic of betalain-producing species [103].

Betalains and anthocyanins appear to be mutually exclusive pigments, as no evidence supports their co-occurrence within the same plant. Both pigment classes fulfil similar biological roles by contributing vivid colouration to flowers and fruits and by accumulating in response to abiotic stresses, suggesting the convergent evolution of function and possibly similar regulatory frameworks [103,108]. Early studies of the leaves of *Phytolacca americana* (Phytolacaceae, Caryophyllales) demonstrated that the gene encoding DOD is transcriptionally regulated by MYB and bHLH transcription factors that bind to specific motifs within the promoter regions [109]. As mentioned above, these transcription factors form the well-known MBW complex (MYB-bHLH-WDR), which regulates flavonoid and anthocyanin biosynthesis [110]. However, MYB1, the first betalain-specific MYB regulator identified in *B. vulgaris*, does not interact with bHLH proteins, distinguishing it from the typical anthocyanin-associated MYBs. This divergence suggests that although these MYBs may share a common evolutionary origin, the regulatory mechanisms governing betalain and anthocyanin biosynthesis have functionally diverged [111].

Comparative genetic studies have identified anthocyanin biosynthetic enzymes encoding genes in betalain-producing taxa; however, they do not result in anthocyanin accumulation. While early biosynthetic genes, such as *CHS*, are expressed throughout all tissues, *DFR* and *ANS* are barely expressed in most mature plant organs [112,113]. These observations hint at the loss of key transcriptionally and/or post-transcriptionally regulatory factors for anthocyanin production in the core Caryophyllales [114]. Phylogenetic analyses support the hypothesis of a single origin of betalain pigmentation early in the evolution of Caryophyllales, with subsequent loss in some lineages that reverted to anthocyanin production [108]. Despite these advances, the molecular mechanisms maintaining the strict mutual exclusivity of betalains and anthocyanins remain incompletely resolved [107]. Despite their distinct biosynthetic origins, the striking functional similarity between these pigment classes represents a compelling example of evolutionary convergence in plant specialized metabolism [115]. The persistence of this mutual exclusivity across evolutionary time suggests strong selective pressures maintaining discrete pigment biosynthetic programmes within each lineage (Figure 2).

Recently, the betalain biosynthetic pathway has been engineered using CRISPR-based strategies to produce novel flower colouration. In a study by Nishihara et al. (2023) [116], conducted on *Gentiana triflora* (Gentianaceae, Gentinales), CRISPR-mediated knockout was applied to supress *DFR* and consequently anthocyanins production. Additionally, the expression of two key betalain biosynthetic genes (CYP76AD6 from *Beta vulgaris* and DOD from *Mirabilis jalapa* (Nyctaginaceae, Caryophyllales)) were introduced, leading to the biosynthesis of betaxanthins. As a result, the genetically modified plants developed vivid yellow flowers, demonstrating a remarkable shift from their native blue colouration. Similarly, in *Torenia fournieri* (Scrophulariaceae, Lamiales), the introduction of *CYP76AD6*, *DOD*, and *UGT* genes led to betacyanin accumulation, shifting the flower colour from purple to reddish [117]. Together, these studies exemplify how the integration of genome editing and transgenic approaches can expand the repertoire of floral pigmentation, offering new avenues for ornamental plant improvement and diversification.

## 5. Integrative Overview of Flower Colour Change

Floral colouration is often dynamic, changing markedly during flower development. Such temporal shifts in pigmentation are driven by pigment degradation and biosynthesis, changes in vacuolar pH, metal ion interactions and co-pigmentation processes [118]. This may involve the whole flower, only the centre, the corolla tube, the nectary/hypanthium, the nectar guide/banner petal spot, selected petals, the petal appendages, the androecium, or the gynoecium [118]. These transitions are not random nor merely symptomatic of senescence; rather, they serve as adaptive signals in pollination ecology, providing visual cues about floral receptivity. In this sense, these changes serve as critical ecological signals, communicating the floral reward status and reproductive phase to pollinators, thus directly influencing pollinator behaviour, visitation efficiency, and ultimately plant reproductive success [119]. In some species—such as *Weigela middendorffiana*, *W. japonica* (Caprifoliaceae, Dipsacales), and *Pedicularis monbeigiana* (Orobanchaceae, Lamiales)— the provision of abundant nectar is closely associated with high pollen viability and stigma receptivity at the initial floral stage; all three features tend to decline following the flower colour change [120,121,122,123]. However, floral colour change does not always correlate with pollen viability. For example, in *Pleroma raddianum* (Melastomataceae, Myrtales), white and pink flowers showed similar pollen viability and stigma receptivity; however, pollinators preferred white flowers but visited pink ones later in the season, suggesting that colour change may serve to broaden visitor diversity [124].

Flower colour change from opening to senescence is a natural phenomenon, widespread in angiosperms, but it is an uncommon strategy limited to a few numbers of species in each order (Figure 2 and Supplementary Table S1). Out of more than 350 thousand vascular plant species [125], only 151 have been described to exhibit this floral behaviour. After the Weiss (1995) [118] publication, we found 25 new species displaying whole-flower colour change. These 151 species belong to 55 families, representing 24 orders according to APG IV classification.

The diversity of lineages exhibiting flower colour change is broadly distributed across the angiosperm phylogeny, encompassing both monocotyledons and eudicotyledons (Supplementary Table S1). However, there is a notable concentration of species with anthocyanin-based colour changes within the orders Fabales, Lamiales, and Myrtales—all belonging to the eudicots. This taxonomic coverage underscores that floral colour change has evolved multiple times independently, suggesting strong selective pressures favouring this trait. This pattern may reflect not only evolutionary trends but also historical sampling biases and increased research efforts focused on clades of economic or ornamental importance. Figure 2 also reinforces that the occurrence of betalains is restricted to the core Caryophyllales lineage, where they are mutually exclusive with anthocyanins, supporting the long-standing hypothesis of biochemical exclusion between these two pigment classes. The inclusion of a detailed phylogeny of core Caryophyllales in the side panel further highlights this separation.

**Figure 3:**
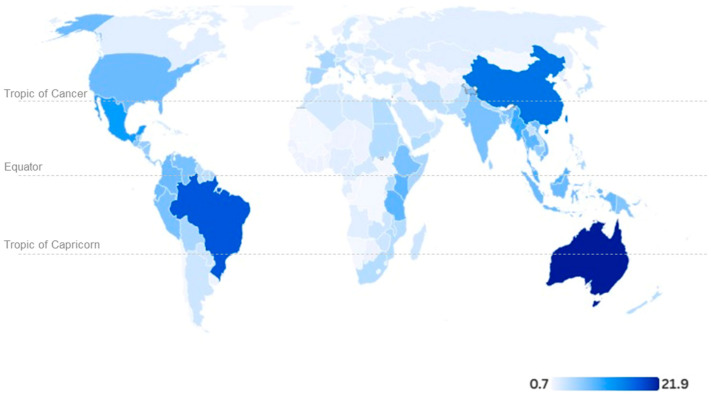
Worldwide native habitat distribution of species displaying flower colour change. Data are shown in percentage with respect to the 151 identified species. The native habitat was retrieved from Taxonomic Name Resolution Services v5.3.1 and Weiss (1995) [118].

Regarding the native habitat distribution, the intertropical region holds the highest number of species presenting floral colour change (Figure 3). Australia accounts for the greatest proportion (33 spp., representing 21.9%), followed by Brazil (28 spp., 17.9%) and China (24 spp., 15.9%). North of the Tropic of Cancer, China and the United States host the largest number of such species (Figure 3). It is important to highlight that this is based on available records, which may be underestimated.

**Figure 4:**
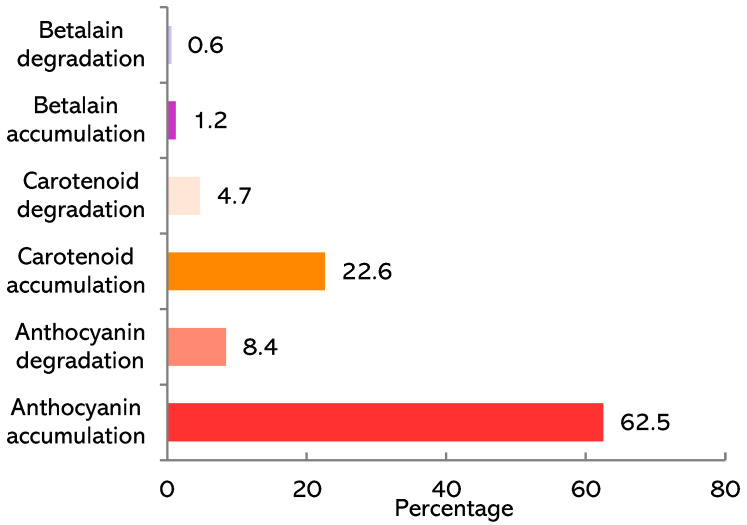
Pigments involved in flower colour change from opening to senescence. Numbers indicate the percentage of each pigment.

Of the species whose flowers change colour from opening to senescence, the majority produce anthocyanins, with the colour shift resulting from pigment accumulation (Figure 4); for 18 species only, the mechanism underlying this phenotype has been elucidated (bolded in Supplementary Table S1).

In *Viola cornuta* (Violaceae, Malpighiales), the flower transitions from white to purple is driven by the temporal upregulation of anthocyanin biosynthetic genes—*CHS*, *DFR*, and *ANS*—regulated by R2R3-MYB transcription factors. This induction is triggered by environmental cues, such as light and pollen deposition on the stigma, leading to anthocyanin accumulation within 24 h post-pollination [126,127]. A similar mechanism occurs in *Nicotiana mutabilis* (Solanaceae, Solanales), where the petal colour changes from white through pink to red, was associated with the *CHS* expression, upregulated by ethylene production, which also accelerates petal senescence and volatile emission [128].

Likewise, in *Pleroma raddianum* (Melastomataceae, Myrtales), the colour change from white to pink is orchestrated by the coordinated expression of key flavonoid biosynthetic genes. From buds to flower white stage, *PAL* upregulation supplies phenolic precursors, while reduced *FLS* expression results in low flavonol levels. The strong induction of *CHS* and *ANS* at the light pink stage yields anthocyanin accumulation, driving the colour shift [129]. Also, in *Gossypium barbadense* and *G. hirsutum* (Malvaceae, Malvales), the transition from cream to pink is associated with the post-anthesis enhancement of the *ANR* biosynthetic genes [130]. In *Combretum indicum* (Combretaceae, Myrtales), the shift from white to red is the consequence of the accumulation of cyanidin and delphinidin derivatives. Comparative transcriptomics data revealed co-induction of anthocyanin and volatile biosynthetic genes, indicating metabolic coordination between colour change and scent production [131].

Interestingly, several transcription factors associated with flower colour change regulation have been identified. During flowering in *Nymphaea atrans* (Nymphaeaceae, Nymphaeales), petal colour shifts from white to deep pink are driven by the marked anthocyanin accumulation. This change is regulated by the coordinated upregulation of *CHS*, *CHI*, *F3H*, *F3′H*, *F3′5′H*, *DFR*, *ANS*, and *UGT*. Three MYB transcription factors (MYB-1, MYB-2, and MYB-3) were identified as putative regulators of these structural genes, showing expression patterns that peaked at specific stages of flowering, consistent with anthocyanin accumulation [132]. In *Chrysanthemum morifolium* (Asteraceae, Asterales) at the blooming stage, MYB7 physically interacts with bHLH2, avoiding its interaction with MYB6 and blocking *DFR* expression. The MYB7 downregulation at post-flowering stage releases MYB6 and bHLH heterodimerization, promoting anthocyanin biosynthesis [133]. Similarly, in *Gloriosa superba* (Colchicaceae, Liliales), flowers deepen from yellow to red through the sequential accumulation of cyanidin-3,5-*O*-diglucoside, cyanidin-3-*O*-glucoside, and pelargonidin-3-*O*-glucoside. Transcriptome analysis reveals a strong correlation in the expression profile between NAC and WRKY TFs and several flavonoid biosynthetic genes [134].

Beyond transcriptional regulation, multifactorial control involving metabolic and hormonal pathways contributes to floral colour modulation. In *P. raddianum*, for instance, primary metabolites, such as soluble sugars and organic acids (e.g., succinic and malic acids), also influence anthocyanin accumulation, underscoring the complexity of metabolic integration underlying the control of flower pigmentation [129]. A more intricate scenario is evident in *Hibiscus mutabilis* (Malvaceae, Malvales) where flowers transition from white to pink to red within a single day. Integrated transcriptomic and metabolomic analyses identified over 180 upregulated genes and a significant accumulation of amino acids (*L*-isoleucine, *L*-methionine), fatty acids, and secondary metabolites correlating with progressive colour deepening. This regulation involves anthocyanin biosynthesis, sulphur, and methionine metabolism, suggesting a multi-layered control involving both primary and specialized metabolic pathways [135,136].

In addition to biosynthetic regulation, anthocyanin degradation also plays a pivotal role in floral colour transitions in several species. A classic example is *Brunfelsia pauciflora* (Solanaceae, Solanales), where petals fade from deep purple to white within a few days post-anthesis, due to the activity of a BASIC VACUOLAR PEROXIDASE [137,138,139]. An analogous mechanism was described in *Nelumbo nucifera* (Nelumbonaceae, Proteales). From the third day after anthesis, anthocyanins fading occurs as a result of the increased vacuolar pH and peroxidase activity [140]. Similarly, in *Malus hupehensis* (Rosaceae, Rosales), the transition from red to white during flower development is primarily driven by a decrease in cyanidin-3-galactoside content, associated with the downregulation of *PAL*, *CHS*, *CHI*, *DFR*, *FLS*, *ANS*, and *UGT.* Although no functional studies were performed, the negative correlation between MYB10 and MYB12 and the positive correlation between MYB6, bHLH33, and WD40 TF expression with colour bleaching suggest their inductive and repressing activity, respectively [141]. Finally, hybrid cultivars of *Paeonia* (Paeoniaceae, Saxifragales) undergo a striking flower colour transition from coral to pink and to pale yellow over approximately 5 to 7 days. Guo et al. (2019) [142] reported that this progressive fading results from a marked reduction in anthocyanin content, particularly cyanidin and peonidin derivatives. The decrease in pigmentation correlates with a significant downregulation of anthocyanin biosynthetic genes (*CHS*, *CHI*, *F3H*, *DFR*, *ANS*, and *UGT*) as the flowers mature. Moreover, transcription factors, such as MYB, bHLH, and the light-responsive HY5 and PIF3, were identified as potential upstream regulators influencing gene expression. These findings suggest that the floral colour fading in *Paeonia* hybrids is primarily governed by transcriptional repression of anthocyanin biosynthesis, integrated with light signalling.

Although anthocyanins are the most frequently associated pigments with flower colour changes, carotenoids also play a significant role in this process. In species such as *Tagetes erecta* (Asteraceae, Asterales), petal colour deepens from pale to vibrant yellow due to the upregulation of *DXS* and *PSY* [82]. In contrast, flower colour lightening can result from the downregulation of carotenoid biosynthetic genes, as observed in *Sandersonia aurantiaca* (Colchicaceae, Liliales), where the reduction in *PDS* expression leads to flower paling [143]. In *Lilium brownii* (Liliaceae, Liliales), the flower colour change from yellowish cream to white was linked to the upregulation of the degrading enzyme *CCD* [144].

Moreover, recent studies have revealed that, beyond transcriptional regulation, epigenetic mechanisms also contribute to flower colour changes. Functional studies in *Xanthoceras sorbifolium* (Sapindaceae, Sapindales) demonstrated that DNA methylation of a MYB gene promoter can modulate floral colour transitions from yellow to pink by altering the expression of *CHS*, *DFR*, and *ANS* (Lu et al., 2022). Specifically, the reduction in the CHH methylation level in a transposable element on the promoter of an R2R3-MYB encoding gene leads to anthocyanin accumulation during petal development. This epigenetic regulation is tightly associated with developmental stage-specific methylation dynamics and downregulation of DNA methyltransferases [66].

## 6. Conclusions

Flower colour change in angiosperms represents a complex trait shaped by multiple selective pressures, integrating diverse biochemical pathways, regulatory networks, and ecological interactions. While significant advances have been made in elucidating the transcriptional regulation of pigment biosynthesis, particularly in model species, important gaps remain. Notably, the roles of epigenetic modifications—such as DNA methylation and histone modifications—and post-translational mechanisms like protein phosphorylation, ubiquitination, and complex assembly are still poorly understood. Expanding research beyond traditional models to include non-model species, particularly those with dynamic colour transitions, will be essential to uncover the full evolutionary and functional diversity of flower colour change. Future studies integrating multiomics approaches, genome editing tools, and ecological analyses will provide deeper insights into how these intricate regulatory layers interact to shape floral phenotypes across angiosperms.

## Data Availability

This review includes original figures created by the authors and a supplementary table compiling published data from the literature. All sources are cited, and the compiled information is available in the Supplementary Material.

## Supplementary Materials

The following supporting information can be downloaded at https://www.mdpi.com/article/10.3390/plants14142185/s1, Table S1: Angiosperms that display color change along floral development. Reviewed and updated from [118].

## Abbreviations

The following abbreviations are used in this manuscript:

4CL	4-CUMAROIL-COA LIGASE
AACT	ANTHOCYANIDIN ACYL TRANSFERASE
ACT	ACYL TRANSFERASE
ADH	AROGENATE DEHYDROGENASE
ADT	AROGENATE DEHYDRATASE
Am	*Antirrhinum majus*
ANR	ANTHOCYANIDIN REDUCTASE
ANS	ANTHOCYANIDIN SYNTHASE
bHLH	BASIC HELIX-LOOP-HELIX transcription factor
C4H	CINNAMATE 4-HYDROXYLASE
*CCD*	*CAROTENOID CLEAVAGE DIOXYGENASE*
cDOPA	cyclo-DOPA
CHI	CHALCONE ISOMERASE
CHS	CHALCONE SYNTHASE
CI	CAROTENE ISOMERASE
Cm	*Chrysanthemum morifolium*
CoA	Coenzyme A
CRISPR	Clustered Regularly Interspaced Short Palindromic Repeats
CYP76AD	CYTOCHROME P450-TYPE GENE
DFR	DIHYDROFLAVANOL 4-REDUCTASE
DMAPP	dimethylallyl diphosphate
DO	DIPHENOL OXIDASE
DOD	DOPA DIOXYGENASE
DXP	1-deoxy-D-xylulose-5-P
DXS	DXP SYNTHASE
E4P	erythrose 4-phosphate
F3′5′H	FLAVANONE 3′5′-HYDROXYLASE
F3′H	FLAVANONE 3′-HYDROXYLASE
F3H	FLAVANONE 3-HYDROXYLASE
FLS	FLAVONOL SYNTHASE
FS	FLAVONE SYNTHASE
GA3P	glyceraldehyde 3-phosphate
GDS	GPP SYNTHASE
GGDS	GERANYLGERANYL DIPHOSPHATE SYNTHASE
GGPP	geranylgeranyl diphosphate
GPP	geranyl diphosphate
HAT	PHENYLPYRUVATE AMINOTRANSFERASE
IFS	ISOFLAVONE SYNTHASE
IPP	isopentenyl diphosphate
IPPI	ISOPENTENYL DIPHOSPHATE ISOMERASE
IspH	4-HYDROXY-3-METHYLBUT-2-ENYL DIPHOSPHATE REDUCTASE
LCYβ	LYCOPENE β-CYCLASE
LCYε	LYCOPENE ε-CYCLASE
LD	Linear dichroism
L-DOPA	dihydroxyphenylalanine
Ls	*Lilium* sp.
MAL	Malonate pathway
MEP	methylerythritol phosphate pathway
MEV	Mevalonate pathway
Ml	*Mimulus lewisii*
Mt	*Medicago truncatula*
MYB	MYELOBLASTOSIS transcription factor
NS	NEOXANTHIN SYNTHASE
OMT	O-METHYL TRANSFERASE
OMT	*O*-METHYLTRANSFERASE
OR	ORANGE
Pa	*Petunia axilaris*
PAL	PHENYLALANINE AMMONIA LYASE
PAT	PREPHENATE AMINOTRANSFERASE
PDH	PREPHENATE DEHYDROGENASE
PDS	PHYTOENE DESATURASE
PEP	phosphoenolpyruvate
Ph	*Petunia hybrida*
PIFs	PHYTOCHROME INTERACTING FACTORS
PSY	PHYTOENE SYNTHASE
SDH	SHIKIMATE DEHYDROGENASE
SK	Shikimate pathway
SlRIN	RIPENING INHIBITOR
SnRK1	Sucrose Non-Fermenting 1-Related Kinase 1
TFs	transcription factors
Tg	*Tulipa gesneriana*
UGT	UDP GLYCOSYL TRANSFERASE
UGT	UDP-GLUCOSYLTRANSFERASE
WDR	WD40 repeat domain transcription factor
ZDS	ZETA-CAROTENE DESATURASE
ZE	ZEAXANTHIN EPOXIDASE
β-CO	Β-CAROTENE OXYGENASE
β-RH	β-RING HYDROXYLASE
ε-RH	ε-RING HYDROXYLASE
