# Supplementary material for: Molecular Control of Flower Colour Change in Angiosperms
Source: Plants (Basel). 2025 Jul 15;14(14):2185. doi: 10.3390/plants14142185 (PMC12299958; doi:10.3390/plants14142185)
Supplement: Supplementary file 1 [file plants-14-02185-s001.zip › plants-3703510-supplementary.pdf]

plants

Review

Molecular Control of Flower Colour Change in Angiosperms

Rezende, F.M.[1]*; Rossi, M.[1]; Furlan, C.M.[1]

Department of Botany, Institute of Biosciences, University of São Paulo
* Correspondence: rezendefmd@gmail.com

Supplementary Material

Supplementary Table S1. Angiosperms that display color change along floral development. Reviewed and updated from Weiss 1995.

Taxon: Order	Taxon: Family	Taxon: Specie	Color change[1]	Pigment involved[2]	Native habitat[3]	Reference
Apiales						
	Pittosporaceae					
		Marianthus ringens (J.Drumm. ex Harv.) F.Muell.	Yellow-orange to red	(+) A	Australia	[1]
		Auranticarpa rhombifolia (A.Cunn. ex Hook.) L.W.Cayzer, Crisp & I.Telford	White to orange-yellow	(+) C	Australia	[2]
		Pittosporum undulatum Vent.	White to yellow	(+) C*	Australia	[3]
Asparagales						
	Asparagaceae					
		Beschorneria yuccoides K.Koch	Green to pink	(+) A*	Mexico	[3]
	Orchidaceae					
		Cyrtorchis arcuata (Lindl.) Schltr.	White to yellow	(+) C	Kenya, Tanzania, Uganda, Ethiopia, Malawi, Mozambique, Zambia, Zimbabwe, South Africa, Benin, Ghana, Guinea, Guinea-Bissau, Ivory Coast, Liberia, Nigeria, Senegal, Sierra Leone, Togo, Burundi, Cameroon, Central African Republic, Gabon, Rwanda, Democratic Republic of the Congo	[4]
		Epidendrum ciliare L.	White to pale yellow	(+) C	Mexico, Belize, Costa Rica, El Salvador, Guatemala, Honduras, Nicaragua, Panama, Brazil, French Guiana, Guyana, Suriname, Venezuela, Bolivia, Colombia, Ecuador, Peru	[3]

	Epidendrum paniculatum Ruiz & Pav.	White- to yellow	(+) C	Brazil, Costa Rica, Panama, French Guiana, Guyana, Suriname, Venezuela, Argentina, Paraguay, Bolivia, Colombia, Ecuador, Peru	[3]
	Epidendrum stamfordianum Bateman	White to pale yellow	(+) C	Mexico, Belize, Costa Rica, El Salvador, Guatemala, Honduras, Nicaragua, Panama, Venezuela, Colombia	[3]
	Malaxis seychellarum (Kraenzl.) Summerh.	Green to brown	(+) A, (+) C	Seychelles	[3]
	Vanda sp.	Lavender to white	(-) A*	China, Japan, South Korea, India, Bangladesh, Nepal, Sri Lanka, Cambodia, Laos, Myanmar, Thailand, Vietnam, Malaysia, Indonesia, Philippines, Papua New Guinea, Solomon Islands, Australia	[5]
	Ypsilopus longifolius (Kraenzl.) Summerh.	White to yellow-green	(+) C	Kenya, Tanzania	[4]
Asterales					
Asteraceae					
	***Chrysanthemum morifolium* Ramat.**	**White to pink**	**(+) A***	**China, Japan**	**[6]**
	***Tagetes erecta* L.**	**White to dark orange**	**(+) C***	**Mexico**	**[7]**
Campanulaceae					
	Lobelia excelsa Bonpl.	Orange to red	(+)A	Southern America Southern South America Chile CentralChile North	[3]
Brassicales					
Brassicaceae					
	Erysimum bicolor (Hornem.) DC.	White to purple	(+) A	Portugal	[3]

		Erysimum scoparium (Brouss. ex Willd.) Wettst.	White to purple	(+) A	Spain	[3]
		Erysimum mutabile Boiss. & Heldr.	White to lavender	(+) A	Greece	[3]
		Iberis linifolia L.	White to lavender	(+) A	Germany, Switzerland, Italy, Croatia, France, Spain	[3]
		Lobularia maritima (L.) Desv.	White to purple	(+) A*	Algeria, Egypt, Libya, Morocco, Tunisia, Israel, Jordan, Syria, Lebanon, Turkey, Cyprus, Italy, France, Portugal, Spain	[3]
Caryophyllales						
	Aizoaceae					
		Carpobrotus edulis (L.) N.E.Br.	Pale yellow to pink	(+) B*	Brazil	[3]
	Cactaceae					
		Opuntia phaeacantha Engelm.	Yellow to orange	(+) B	United States**	[3]
	Polygonaceae					
		Polygonum emondi Meisn.	Red to whitish	(-) B	India, Nepal, Bhutan, China, Pakistan	[3]
Dipsacales						
	Caprifoliaceae					
		Diervilla lonicera Mill.	Yellow to orange	(+)A, (+) C*	Canada, United States	[8]
		Lonicera hildebrandiana Collett & Hemsl.	White to yellow-orange	(+)A, (+) C	China, India, Laos, Myanmar, Thailand, Vietnam	[3]
		*Lonicera japonica T*hunb.	White to yellow	(+)A, (+) C*	Japan**	[3]
		Lonicera morrowii A.Gray	White to yellow	(+)A, (+) C	China, Japan, South Korea, North Korea	[3]

		Lonicera periclymenum L.	White to yellow	(+)A, (+) C	Morocco, Austria, Belgium, Germany, Netherlands, Poland, Switzerland, Denmark, United Kingdom, Ireland, Norway, Sweden, Albania, Greece, Italy, Croatia, France, Portugal, Spain	[3]
		Lonicera tatarica L.	White to yellow	(+)A, (+) C	Armenia, Transcaucasus, China, Kazakhstan, Kyrgyzstan, Mongolia, Russia	[3]
		Patrinia villosa Juss.	Yellow to white	(+)A, (-) C	China**	[3]
		Weigela decora (Nakai) Nakai	Pale yellow to pink	(+)A, (+) C	Asia-Temperate *Eastern Asia* Japan	[3]
Ericales						
	Ericaceae					
		Brachyloma preissii Sond.	White to red	(+) A	Australia	[1]
	Polemoniaceae					
		Cobaea scandens Cav.	Whitish to purple	(+) A	Mexico	[3]
Fabales						
	Fabaceae					
		Acmispon glaber (Vogel) Brouillet	Yellow to deep orange	(+) C	Mexico, United States	[3]
		Argyrolobium fischeri Taub.	Bright yellow to red	(+) A	Kenya, Tanzania, Uganda, Ethiopia, Sudan, Angola, Malawi, Mozambique, Zambia, Rwanda, Democratic Republic of the Congo (formerly Zaire)	[4]
		Bauhinia bidentata Jack	Yellow to red	(+)A, (+) C	Thailand	[9]
		Cadia purpurea(G.Piccioli)Aiton	White to dark pink	(+) A	Kenya, Eritrea, Ethiopia, Somalia, Oman, Saudi Arabia, Yemen	[10]
		Castanospermum australe A.Cunn & C.Fraser ex Hook	Yellow to red	(+) A	Australia	[11]

Colutea multiflora Ali	Yellow to pink-red	(+) A	Nepal	[12]
Dichrostachys cinerea (L.) Wight & Arn.	Pink to white	(-) A	Kenya, Tanzania, Uganda, Cape Verde, Chad, Eritrea, Ethiopia, Somalia, Sudan, Angola, Malawi, Mozambique, Zambia, Zimbabwe, Botswana, Namibia, Eswatini (formerly Swaziland), Benin, Burkina Faso, Ghana, Guinea, Guinea-Bissau, Ivory Coast, Liberia, Mali, Mauritania, Niger, Nigeria, Senegal, Sierra Leone, Togo, Burundi, Cameroon, Central African Republic, Congo, Gabon, Rwanda, Democratic Republic of the Congo (formerly Zaire), Oman, Saudi Arabia, Yemen, India, Sri Lanka, Myanmar, Malaysia, Indonesia, Australia, South Africa	[13]
Intsia bijuga (Colebr.)Kuntze	White to pink	(+)A, (+) C	Tanzania, Chagos Archipelago, Madagascar, Mauritius, Seychelles, China, Bangladesh, India, Sri Lanka, Cambodia, Myanmar, Thailand, Vietnam, Malaysia, Indonesia, Philippines, Papua New Guinea, Solomon Islands,Australia,Fiji, fench pacific island, Samoa, Tonga, Vanuatu	[3]
Lotononis eriantha Benth.	Yellow to orange-red	(+) A, (+) C	South Africa**	[13]
Lotononis laxa Eckl. & Zeyh	Yellow to orange	(+) C	Kenya, Tanzania, Uganda, Ethiopia, Malawi, Zimbabwe, South Africa, Lesotho, Eswatini (formerly Swaziland)	[13]

	Lotus corniculatus L.	Yellow to deep orange	(+) A, (+) C	Kenya, Tanzania, Uganda, Ethiopia, Malawi, Zimbabwe, Eritrea, Sudan, Algeria, Egypt, Libya, Morocco, Tunisia, Yemen, Kazakhstan, Tajikistan, Afghanistan, Cyprus, Iran, Iraq, Lebanon, Syria, Palestine, Turkey, Myanmar, Nepal, Pakistan, Belarus, Russia, Ukraine, Austria, Belgium, Czech Republic, Germany, Hungary, Netherlands, Poland, Switzerland, Denmark, Finland, Faroe Islands, United Kingdom, Ireland, Norway, Sweden, Albania, Bulgaria, Greece, Italy, Romania, Serbia, France, Portugal, Spain	[14]
	Lotus pedunculatus	Yellow to red	(+) A	Algeria, Egypt, Libya, Morocco, Tunisia, Turkey, Belarus, Ukraine, Austria, Belgium, Czech Republic, Germany, Netherlands, Poland, Switzerland, Denmark, United Kingdom, Ireland, Sweden, Albania, Bulgaria, Greece, Italy, Romania, Serbia, France, Portugal, Spain	[3]
	Mimosa rubicaulis Lam.	Pink to white	(-) A	India, Bangladesh, Nepal, Pakistan, Myanmar	[12]
	Pearsonia sessilifolia (Harv.) Dummer	Yellow to red	(+) A	South Africa	[13]
	Pediomelum esculentum (Pursh) Rydb.	Lavender to pale yellow	(-) C	United States	[15]
	Saraca declinata Miq.	Pale yellow to red	(+)A, (+) C	Bangladesh, Cambodia, Laos, Myanmar, Thailand, Vietnam, Malaysia, Indonesia, Papua New Guinea	[9]
	Saraca indica L.	Pale orange to red	(+)A, (+) C	Thailand, Laos, Vietnam, Malaysia, Indonesia	[3]
	Saraca cauliflora Baker	Yellow to deep orange-red	(+)A, (+) C	Cambodia, Myanmar, Thailand, Vietnam, Malaysia, Indonesia, Papua New Guinea	[16]

		Acmispon tomentosus (Hook. & Arn.) Govaerts	Yellow to red-orange	(+) A, (+) C	Mexico, United States	[3]
		Trifolium stellatum L.	White to deep pink	(+) A	Algeria, Egypt, Libya, Morocco, Tunisia,Cyprus, Iran, Iraq, Lebanon, Syria, Palestine, Turkey,Albania, Greece, Italy, Serbia, France, Portugal, Spain	[3]
	Polygalaceae					
		Polygala cruciata L.	White to pink	(+) A	United States, Canada	[17]
		Polygala curtissii A. Gray	White and yellow to pink	(+) A	United States	[17]
Gentianales						
	Rubiaceae					
		Canthium lucidum R.Br.	White to yellow	(+) C	Ethiopia	[18]
		Glionnetia sericea (Baker) Tirveng.	White to dark red	(+) A	Seychelles	[3]
		Hamelia patens Jacq.	Orange to red	(+) A	Mexico, United States, Brazil, Argentina, Paraguay, Bolivia, Colombia, Ecuador, Peru, Bahamas, Cuba, Dominican Republic, Haiti, Jamaica, Puerto Rico, Trinidad and Tobago, Belize, Costa Rica, El Salvador, Guatemala, Honduras, Nicaragua, Panama,French Guiana, Guyana, Venezuela, Brazil	[3]
		Ixora javanica (Blume) DC.	Yellow-orange to red	(+) A	Bangladesh, Cambodia, Myanmar, Thailand, Vietnam, Malaysia, Indonesia	[16]
	Apocynaceae					
		Cynanchum revoilii (Franch.) Khanum & Liede	White to yellow-bronze	(+) C, pH	Kenya, Tanzania, Djibouti, Ethiopia, Somalia	[4]
		Oxypetalum coeruleum(D. Don ex Sweet) Decne.	Pale blue to pink	(+) C, pH	Brazil, Uruguay	[3]

Lamiales				
Acanthaceae				
Asystasia gangetica (L.) T.Anderson	Pale yellow to lavender	(+)A	Bangladesh, India, Sri Lanka, Cambodia, Myanmar, Thailand, Vietnam, Malaysia, Papua New Guinea, Australia	[19]
Avicennia marina (Forssk.) Vierh.	Yellow to orange	(+)A, (-) C*	Kenya, Tanzania, Djibouti, Eritrea, Somalia, Sudan, Egypt, Mozambique, Comoros, Madagascar, Seychelles, Saudi Arabia, Yemen, Oman, China, Iran, Myanmar, Bangladesh, India, Pakistan, Sri Lanka, Thailand, Vietnam, Malaysia, Indonesia, Philippines, Papua New Guinea, Australia, New Zealand, Fench pacific island, Vanuatu	[18]
Crossandra johanninae Fiori	White to lavender	(+)A	Eritrea, Somalia, Saudi Arabia, Yemen	[10]
Bignoniaceae				
Mansoa hymenaea (DC.) A.H.Gentry	Purple to white	(-) A	Mexico, Brazil, Belize, Costa Rica, El Salvador, Guatemala, Honduras, Nicaragua, Panama, French Guiana, Suriname, Venezuela, Colombia, Ecuador, Peru	[20]
Boraginaceae				
Arnebia euchroma (Royle) I.M.Johnst.	Pink to deep purple	(+) A, pH	China, Kazakhstan, Kyrgyzstan, Tajikistan, Turkmenistan, Uzbekistan, Afghanistan, Iran, Nepal, Pakistan	[3]
Heliotropium arbainense Fresen.	Pale cream to deep yellow	(+) C	Ethiopia, Sudan, Egypt, Madagascar, Saudi Arabia, Afghanistan, Iran, Lebanon, Syria, Palestine	[10]
Oreocarya flava A.Nelson	White to yellow	(+) C	United States	[3]

	Pulmonaria officinalis L.	Red to blue	pH	Belarus, Ukraine, Austria, Belgium, Czech Republic, Germany, Hungary, Netherlands, Poland, Switzerland, Denmark, Sweden, Albania, Bulgaria, Greece, Italy, Romania, Serbia, France	[21]
Orobanchaceae					
	Pedicularis monbeigiana Bonati	White to purple	(+) A	China, Myamar	[22]
Plantaginaceae					
	Veronica townsonii Cheeseman	Purple to white	(-) A	New Zealand	[3]
Scrophulariaceae					
	Buddleja marrubiifolia Benth.	Yellow to red	(+) A	Mexico, United States	[3]
Verbenaceae					
	Lantana camara L.	Yellow to red	(+)A, (-) C*	Mexico, Brazil, Aruba, Bahamas, Cayman Islands, Cuba, Dominican Republic, Haiti, Jamaica, Netherlands Antilles, Puerto Rico, Trinidad and Tobago, Venezuela, Belize, Costa Rica, El Salvador, Guatemala, Honduras, Nicaragua, Panama, Guyana, Colombia, Ecuador	[23]
	Lantana viburnoides (Forssk.) Vahl	Yellow to deep pink	(+)A, (-) C*	Kenya, Tanzania, Uganda, Chad, Djibouti, Eritrea, Ethiopia, Somalia, Sudan, Egypt, Angola, Malawi, Mozambique, Zambia, Zimbabwe, Botswana, Niger, Nigeria, Cameroon, Central African Republic, Congo, Rwanda, Democratic Republic of the Congo (formerly Zaire), Oman, Yemen	[10]

Liliales					
Colchicaceae					
	Gloriosa superba **L.**	**Yellow-green to dark purple**	**(+) A***	**Kenya, Tanzania, Uganda, Chad, Ethiopia, Sudan, Angola, Malawi, Mozambique, Zambia, Zimbabwe, Botswana, South Africa, Eswatini, Benin, Burkina Faso, Ghana, Guinea, Guinea-Bissau, Ivory Coast, Liberia, Nigeria, Senegal, Sierra Leone, Togo, Burundi, Cabinda (region of Angola), Cameroon, Central African Republic, Congo, Equatorial Guinea, Gabon, Rwanda, Democratic Republic of the Congo, Madagascar, Seychelles, China, India, Bangladesh, Nepal, Pakistan, Sri Lanka, Cambodia, Laos, Myanmar, Thailand, Vietnam, Malaysia, Indonesia, Philippines.**	**[24]**
	Sandersonia aurantiaca **Hook.**	**pale yellow to deep yellow**	**(+) C***	**South Africa, Eswatini (formerly Swaziland)**	**[25]**
Liliaceae					
	Lilium brownii **F.E.Br. ex Miellez**	**Yellow to white**	**(-) C***	**China**	**[26]**
Melanthiaceae					
	Trillium ovatum Pursh	White to deep pink	(+) A	United States, Canada	[3]
	Zigadenus glaberrimus Michx.	White to pale yellow	(+) C	United States	[3]
Magnoliales					
Myristicaceae					
	Myristica insipida R.Br.	White to purple	(+) A	Australia, Malaysia	[27]
Malphighiales					
Malpighiaceae					
	Byrsonima crassifolia (L.) Kunth	Yellow to orange	(+) A	Costa Rica**	[3]

		Byrsonima microphylla A.Juss.	Yellow to red	(+) A	Brazil	[28]
		Byrsonima gardnerana A.Juss.	Yellow to red	(+) A	Brazil	[28]
		Heteropterys alternifolia W.R. Anderson	Yellow to red	(+) A	Brazil	[28]
		Tetrapterys phlomoides (Spreng.) Nied.	Yellow to orange	(+) A	Brazil	[3]
	Passifloraceae					
		Passiflora sp.	White to yellow	(+) C	Australia, New Zealand	[3]
	Rhizophoraceae					
		Ceriops tagal (Perr.) C.B.Rob.	White to orange	(+) A	Kenya, Tanzania, Djibouti, Somalia, Mozambique, Comoros, Madagascar, Seychelles, China, Bangladesh, India, Pakistan, Sri Lanka, Cambodia, Myanmar, Thailand, Vietnam, Malaysia, Indonesia, Philippines, Papua New Guinea, Australia, New Zealand, fench pacific island, Vanuatu	[18]
	Violaceae					
		***Viola cornuta* L.**	**White to purple**	**(+)A***	**France, Spain**	[29,30]
Malvales						
	Malvaceae					
		Brachychiton discolor F.Muell.	White to purple	(+) A	Australia	[3]
		***Gossypium barbadense* L.**	**Cream to pink**	**(+) A***	**Colombia, Ecuador, Peru**	[31]
		***Gossypium hirsutum*L.**	**Cream to pink**	**(+) A***	**Mexico, Brazil, Aruba, Netherlands Antilles, Costa Rica, El Salvador, Guatemala, Honduras, Nicaragua, French Guiana, Guyana, Suriname, Venezuela, Colombia, Ecuador, Fiji, Samoa, French,French Polynesia**	[3,31]

***Hibiscus mutabilis* L.**	**White to pink**	**(+) A ***	**China**	**[32–34]**
Hibiscus tilliaceous L.	Yellow to orange-red	(+) A	Central African Republic, Panama, South Africa, Gabon	[3]
Lasiopetalum behrii	Pale yellow to pink	(+) A	Australia	[35]
Malvaviscus arboreus Cav.	Red to pink	(-) A*	United States, Peru, Brazil	[36]
Thespesia populnea (L.) Sol. ex Corrêa	Yellow to purple	(+) A*	Kenya, Tanzania, Djibouti, Eritrea, Sudan, Mozambique, Comoros, Madagascar, Mauritius, Seychelles, Oman, China, Bangladesh, India, Maldives, Sri Lanka, Cambodia, Myanmar, Thailand, Vietnam, Malaysia, Indonesia, Philippines, Papua New Guinea, Australia, Fiji, Nauru, fench pacific island, Niue, Samoa, Tonga, Vanuatu, Wallis and Futuna, United States, Caroline Islands, Marianas, Marshall Islands, Wake Island, Line Islands, France, Pitcairn Islands, French Polynesia, Tubuai Islands	[37]
Thymelaeaceae				
Edgeworthia chrysantha Lindl.	Yellow to white	(-) A, (-) C	China, Myanmar	[3]
Pimelea ferruginea Labill.	Pink to white	(-) A, (-) C	Australia	[3]
Myrtales				
Combretaceae				
Combretum farinosum Kunth	Green to orange	(+) A	Mexico, Costa Rica, El Salvador, Guatemala, Honduras, Nicaragua	[38]
***Combretum indicum* (L.) DeFilipps**	**White to deep red**	**(+) A**	**Tanzania, China, Bangladesh, India, Nepal, Sri Lanka, Cambodia, Laos, Myanmar, Thailand, Vietnam, Malaysia, Indonesia, Philippines, Papua New Guinea, Australia**	**[39]**

Melastomataceae					
	Andesanthus lepidotus (Humb. & Bonpl.) P.J.F.Guim. & Michelang.	Magenta to purple	(+) A*	Panama, Venezuela, Colombia, Ecuador, Peru	[3]
	***Pleroma raddianum* Gardner**	**White to dark pink**	**(+) A**	**Brazil**	**[40,41]**
	Pleroma sellowianum (Cham.) P.J.F.Guim. & Michelang.	White to red	(+) A	Brazil	[3]
Myrtaceae					
	Chamelaucium megalopetalum F.Muell. ex Benth.	White to red	(+) A	Australia	[1]
	Darwinia citriodora (Endl.) Benth.	Yellow to red	(+) A	Australia	[42]
	Darwinia fascicularis Rudge	White to red	(+) A	Australia	[42]
	Hypocalymma angustifolium (Endl.) Schauer	White to deep pink	(+) A	Australia	[42]
	Myrtella sp.	White to pink	(+) A	Papua New Guinea, Solomon Islands	[18]
	Verticordia acerosa Lindl.	Yellow to orange-red	(+) A	Australia	[42]
	Verticordia chrysantha Endl.	Yellow to red	(+) A	Australia	[42]
	Verticordia grandiflora Endl.	Yellow to orange	(+) A	Australia	[1]
	Verticordia huegelii Endl.	White to red	(+) A	Australia	[42]
Onagraceae					
	Fuchsia excorticata (G.Forst.) L.f.	Green and black to deep red	(+) A*	New Zealand	[43,44]
	Hauya sp.	White to deep pink	(+) A*	Mexico, Belize, Costa Rica, El Salvador, Guatemala, Honduras, Nicaragua	[3]

		Oenothera epilobiifolia Kunth	Green to orange-red	(+) A	Mexico, Costa Rica, Guatemala, Venezuela, Colombia, Ecuador, Peru	[3]
		Oenothera laciniata Hill	Yellow to orange	(+) A*	Mexico, United States	[45]
		Oenothera stricta Ledeb.	Yellow to orange	(+) A*	Argentina, Chile	[45]
		Oenothera suffrutescens (Ser.) W.L.Wagner & Hoch	White to maroon	(+) A	Mexico, United States, Canada	[3]
		Oenothera tetraptera Cav.	White to pink	(+) A*	Mexico, Costa Rica, Guatemala, Honduras, Venezuela, Bolivia, Colombia, Ecuador, Peru	[45]
	Vochysiaceae					
		Qualea multiflora Mart.	White to pale yellow	(+) C	Brazil, Paraguay, Bolivia, Peru	[3]
Nymphaeales						
	Nymphaeaceae					
		***Nymphaea atrans* S.W.L.Jacobs**	**white to pink**	**(+) A***	**Australia**	**[46]**
		Victoria amazonica (Poepp.) J.C. Sowerby	White to pale purple	(+) A*	Brazil	[3]
		Victoria boliviana Magdalena & L.T.Sm.	White to pale purple	(+) A	Bolivia	[47]
		Victoria cruziana Orbign.	White to pale purple	(+) A	Argentina, Brazil, Paraguay	[47]
Oxalidales						
	Elaeocarpaceae_					
		Aristotelia fruticosa Hook.f.	White to red	(+) A	New Zealand	[3]
		Aristotelia serrata (J.R.Forst. & G.Forst.) Oliv.	White to red	(+) A	New Zealand	[3]

Poales						
	Bromeliaceae					
		Aechmea sp.	Pink to blue	(+)A pH	Mexico, Brazil, Cuba, Dominican Republic, Haiti, Jamaica, Puerto Rico, Trinidad and Tobago, Venezuela, Belize, Costa Rica, El Salvador, Guatemala, Honduras, Nicaragua, Panama, French Guiana, Guyana, Suriname, Argentina, Paraguay, Uruguay, Bolivia, Colombia, Ecuador, Peru	[3]
Proteales						
	Proteaceae					
		Banksia ilicifolia R.Br.	Yellow to red	(+) A	Australia	[48]
		Leucospermum oleaefolium R. Br.	Yellow to orange-red	(+) A	South Africa	[49]
	Nelumbonaceae					
		***Nelumbo nucifera* Gaertn.**	**Pink to white**	**(-) A***	**Armenia, China, Japan, Korea, Russia, Iran, Bangladesh, India, Nepal, Pakistan, Sri Lanka, Cambodia, Laos, Myanmar, Thailand, Vietnam, Malaysia, Indonesia, Philippines, Papua New Guinea, Australia, Ukraine**	**[50]**
Rosales						
	Rhamnaceae					
		Cryptandra amara Sm.	White to red	(+) A	Australia	[3]
	Rosaceae					
		Rosa chinensis f. *mutabilis* (Correvon) Rehder	Yellow to deep pink	(+) A*	China**	[3,51]
		***Malus hupehensis* (Pamp.) Rehder**	**Red to white**	**(-) A***	**China**	**[52]**

Santalales				
Loranthaceae				
Phragmanthera dshallensis (Engl.) M.G.Gilbert	Yellow to red	(+) A	Kenya, Tanzania, Uganda, Ethiopia, Somalia, Malawi, Zambia	[53]
Phragmanthera regularis (Steud. ex Sprague) M.G. Gilbert	Yellow to red	(+) A	Kenya, Tanzania, Ethiopia, Yemen	[4]
Santalaceae				
Santalum freycinetianum F. Phil.	Greenish-yellow to red	(+) A*	United States	[54]
Santalum haleakalae Hillebr.	White to red	(+) A	United States	[54]
Santalum album L.	White to red	(+) A	Malaysia, Indonesia, Philippines, Australia	[1]
Schoepfiaceae				
Quinchamalium chilense Molina	Yellow to orange	(+) A*	Argentina, Chile, Bolivia, Peru	[55]
Sapindales				
Anacardiaceae–				
Anacardium occidentale L.	White to pink	(+) A	Brazil, Trinidad and Tobago, Guyana, Suriname, Venezuela, Bolivia, Colombia, Ecuador, Peru	[3]
Rutaceae				
Boronia molloyae J.R.Drumm.	Magenta to deep red	(+) A	Australia	[3]
Boronia polygalifolia Sm.	Pale yellow to pinkish	(+) A	Australia**	[3]
Sapindaceae				
***Xanthoceras sorbifolium* Bunge**	**Yellow to pink**	**(+) A***	**China, Korea**	[56]

Saxifragales				
Paeoniaceae				
***Paeonia hybrida* 'Coral Sunset' and 'Pink Hawaiian Coral'**	**Coral to pale yellow**	**(-) A***	**China**	[57]
Saxifragaceae				
Tellima grandiflora (Pursh) Douglas ex Lindl.	White to red	(+) A	United States**	[3]
Solanales				
Convovulaceae				
Ipomoea tricolor Cav.	red to blue	(+) A pH	Mexico	[58]
Solanaceae				
Brugmansia versicolor Lagerh.	Cream to pink	(+) A	Brazil**	[3]
***Brunfelsia pauciflora* (Cham. & Schltdl.) Benth.**	**Purple to white**	**(-) A pH***	**Brazil**	[59–61]
Solanum diploconos (Mart.) Bohs	Violet to yellow-ochre	(-) A, (+) C	Brazil	[62]
Solanum endopogon (Bitter) Bohs	Lavender to greenish-white	(-) A	Brazil	[63]
Solanum sciadostylis (Sendtn.) Bohs	Violet to yellow-ochre	(-) A, (+) C	Brazil	[62]
***Nicotiana mutabilis* Stehmann & Semir**	**White to pink**	**(+) A***	**Brazil**	[64]
Solandra sp.	White to yellow	(+) C	Mexico, Brazil, Cuba, Dominican Republic, Haiti, Jamaica, Puerto Rico, Trinidad and Tobago, Belize, Costa Rica, El Salvador, Guatemala, Honduras, Nicaragua, Panama, French Guiana, Venezuela, Bolivia, Colombia, Ecuador, Peru	[49]
Streptosolen jamesonii (Benth.) Miers	Yellow to deep orange	(+) C	Ecuador, Peru	[3]

[1] Color change described for sepals, petals or both.

[2] Biochemical process (*i.e.* pH or pigments) of flower color change from opening to senescence. (+) A: anthocyanin accumulation, (-) A: anthocyanin degradation; (+) C: carotenoid accumulation, (-) C: carotenoid degradation, (+) B: betalain accumulation, (-) B: betalain degradation. One asterisk indicate that the pigment was experimentally identified, otherwise the pigment class was inferred by colour.

[3] Native habitat was determined by the world flora online (https://wwwworldfloraonlineorg/taxon/wfo-0000223016). Two asterisks indicate species for those that the native habitat was determined by Weiss (1995).

Species for those the molecular mechanism of flower colour change addressed are highlighted in bold.

References

1. Erickson, R. *Flowers and Plants of Western Australia*; Reed, 1973; ISBN 978-0-589-07123-3.
2. Bailey, L.H. *Manual of Cultivated Plants*; New York ;: Macmillan Co., 1924;
3. Weiss, M.R. Floral Color Change: A Widespread Functional Convergence. *American Journal of Botany* **1995**, *82*, 167–185, doi:10.1002/j.1537-2197.1995.tb11486.x.
4. Blundell, M. *Collins Guide to the Wild Flowers of East Africa*; London : Collins, 1987; ISBN 978-0-00-219812-7.
5. Burg, S.P.; Dijkman, M.J. Ethylene and Auxin Participation in Pollen Induced Fading of Vanda Orchid Blossoms. *Plant Physiology* **1967**, *42*, 1648–1650, doi:10.1104/pp.42.11.1648.
6. Wang, Y.; Wang, Y.; Zhou, L.-J.; Peng, J.; Chen, C.; Liu, S.; Song, A.; Jiang, J.; Chen, S.; Chen, F. CmNAC25 Targets CmMYB6 to Positively Regulate Anthocyanin Biosynthesis during the Post-Flowering Stage in Chrysanthemum. *BMC Biology* **2023**, *21*, 211, doi:10.1186/s12915-023-01719-7.
7. Moehs, C.P.; Tian, L.; Osteryoung, K.W.; DellaPenna, D. Analysis of Carotenoid Biosynthetic Gene Expression during Marigold Petal Development. *Plant Mol Biol* **2001**, *45*, 281–293, doi:10.1023/A:1006417009203.
8. Schoen, D.J. Floral Biology of Diervilla Lonicera (Caprifoliaceae). *Bulletin of the Torrey Botanical Club* **1977**, *104*, 234–240, doi:10.2307/2484303.
9. Larsen, K.; Larsen, S.S.; Vidal, J.E. *Flora of Thailand: Leguminosae - Caesalpinioideae*; Forest Herbarium, Royal Forest Department, 1984;
10. Collenette, S. *An Illustrated Guide to the Flowers of Saudi Arabia*; London : Scorpion, 1985; ISBN 978-0-905906-45-4.
11. Macoboy, S. *What tree is that?*; Tiger Books;
12. Larsen, K. Polunin, O. & Stainton, A. 1984. Flowers of the Himalaya. *Nordic Journal of Botany* **1985**, *5*, 64–64, doi:10.1111/j.1756-1051.1985.tb02073.x.
13. Wyk, B.V.; Malan, S. *Field Guide to the Wild Flowers of the Witwatersrand & Pretoria Region: Including the Magaliesberg & Suikerbosrand*; Penguin Random House South Africa, 1988; ISBN 978-0-86977-814-2.
14. Jones, E. C.; Ericson, T. *Floral Color Changes in Deerweed (Lotus Scoparius): Possible Function*; Southern California Botanists, Rancho Santa Ana Botanic Garden: Claremont, CA, 1982; Vol. v.8:no.1-6 (1982), pp. 1–68;.
15. Freeman, C.C.; Schofield, E.K. *Roadside Wildflowers of the Southern Great Plains*; University Press of Kansas, 1991; ISBN 978-0-7006-0448-7.
16. Polunin, I. *Plants and Flowers of Singapore*; Times Editions, 1987; ISBN 978-9971-4-0114-6.
17. Duncan, W.H. (Wilbur H. *Wildflowers of the Southeastern United States*; Athens : University of Georgia Press, 1975; ISBN 978-0-8203-0347-5.
18. www.bibliopolis.com Top End Native Plants by John Brock on Andrew Isles Natural History Books Available online: https://www.andrewisles.com/pages/books/2408/john-brock/top-end-native-plants (accessed on 8 July 2025).
19. Gracie, C. Pollination of Cyphomandra Endopogon Var. Endopogon (Solanaceae) by Eufriesea Spp. (Euglossini) in French Guiana. *Brittonia* **1993**, *45*, 39–46, doi:10.2307/2806859.
20. Barrows, E. Floral Maturation and Insect Visitors of Pachyptera Hymenaea (Bignoniaceae). *Biotropica* **1977**, *9*, 133–134.
21. Müller, H. The Effect of the Change of Colour in the Flowers of "Pulmonaria Offcinalis" upon Its Fertilisers. *Nature* **1883**, *28*, 81–81, doi:10.1038/028081d0.
22. Sun, S.G.; Liao, K.; Xia, J.; Guo, Y.H. Floral Colour Change in Pedicularis Monbeigiana (Orobanchaceae). *Plant Syst. Evol.* **2005**, *255*, 77–85, doi:10.1007/s00606-005-0348-y.
23. Mathur, G.; Ram, H.Y.M. Significance of Petal Colour in Thrips-Pollinated Lentana Camara L. *Annals of Botany* **1978**, *42*, 1473–1476.

24. Sun, Y.; Hu, P.; Jiang, Y.; Li, J.; Chang, J.; Zhang, H.; Shao, H.; Zhou, Y. Integrated Metabolome and Transcriptome Analysis of Petal Anthocyanin Accumulation Mechanism in Gloriosa Superba 'Rothschildiana' during Different Flower Development Stages. *International Journal of Molecular Sciences* **2023**, *24*, 15034, doi:10.3390/ijms242015034.
25. Nielsen, K.M.; Lewis, D.H.; Morgan, E.R. Characterization of Carotenoid Pigments and Their Biosynthesis in Two Yellow Flowered Lines of Sandersonia Aurantiaca (Hook). *Euphytica* **2003**, *130*, 25–34, doi:10.1023/A:1022328828688.
26. Hai, N.T.L.; Masuda, J.; Miyajima, I.; Thien, N.Q.; Mojtahedi, N.; Hiramatsu, M.; Kim, J.-H.; Okubo, H. Involvement of Carotenoid Cleavage Dioxygenase 4 Gene in Tepal Color Change in Lilium Brownii Var. Colchesteri. *J. Japan. Soc. Hort. Sci.* **2012**, *81*, 366–373, doi:10.2503/jjshs1.81.366.
27. Armstrong, J.E.; Irvine, A.K. Floral Biology of Myristica Insipida (Myristicaceae), a Distinctive Beetle Pollination Syndrome. *American Journal of Botany* **1989**, *76*, 86–94, doi:10.1002/j.1537-2197.1989.tb11288.x.
28. Costa, C.B.N.; Costa, J.A.S.; Ramalho, M. Biologia reprodutiva de espécies simpátricas de Malpighiaceae em dunas costeiras da Bahia, Brasil. *Braz. J. Bot.* **2006**, *29*, 103–114, doi:https://doi.org/10.1590/S0100-84042006000100010.
29. Farzad, M.; Griesbach, R.; Weiss, M.R. Floral Color Change in *Viola Cornuta* L. (Violaceae): A Model System to Study Regulation of Anthocyanin Production. *Plant Science* **2002**, *162*, 225–231, doi:10.1016/S0168-9452(01)00557-X.
30. Li, Q.; Wang, J.; Sun, H.-Y.; Shang, X. Flower Color Patterning in Pansy (*Viola × Wittrockiana* Gams.) Is Caused by the Differential Expression of Three Genes from the Anthocyanin Pathway in Acyanic and Cyanic Flower Areas. *Plant Physiology and Biochemistry* **2014**, *84*, 134–141, doi:10.1016/j.plaphy.2014.09.012.
31. Tan, J.; Wang, M.; Tu, L.; Nie, Y.; Lin, Y.; Zhang, X. The Flavonoid Pathway Regulates the Petal Colors of Cotton Flower. *PLOS ONE* **2013**, *8*, e72364, doi:10.1371/journal.pone.0072364.
32. ThriftBooks Malaysian Flowers in Colour Book by H F Chin Available online: https://www.thriftbooks.com/w/malaysian-flowers-in-colour/7847726/ (accessed on 8 July 2025).
33. Zhu, Z.; Zeng, X.; Shi, X.; Ma, J.; Liu, X.; Li, Q. Transcription and Metabolic Profiling Analysis of Three Discolorations in a Day of Hibiscus Mutabilis. *Biology* **2023**, *12*, 1115, doi:10.3390/biology12081115.
34. Yang, Y.; Liu, X.; Shi, X.; Ma, J.; Zeng, X.; Zhu, Z.; Li, F.; Zhou, M.; Guo, X.; Liu, X. A High-Quality, Chromosome-Level Genome Provides Insights Into Determinate Flowering Time and Color of Cotton Rose (Hibiscus Mutabilis). *Front. Plant Sci.* **2022**, *13*, doi:10.3389/fpls.2022.818206.
35. Costermans, L.F. *Native Trees and Shrubs of South-Eastern Australia*; Rigby, 1983; ISBN 978-0-7270-1799-4.
36. Gottsberger, G. Colour Change of Petals in Malvaviscus Arboreus Flowers. *Acta Botanica Neerlandica* **1971**, *20*, 381–388, doi:10.1111/j.1438-8677.1971.tb00723.x.
37. Mabberley, D.J. *The Plant-Book: A Portable Dictionary of the Vascular Plants*; Cambridge University Press, 1997; ISBN 978-0-521-41421-0.
38. Schemske, D.W. Floral Ecology and Hummingbird Pollination of Combretum Farinosum in Costa Rica on JSTOR Available online: https://www.jstor.org/stable/2387968?seq=1 (accessed on 8 July 2025).
39. Ghissing, U.; Kutty, N.N.; Bimolata, W.; Samanta, T.; Mitra, A. Comparative Transcriptome Analysis Reveals an Insight into the Candidate Genes Involved in Anthocyanin and Scent Volatiles Biosynthesis in Colour Changing Flowers of Combretum Indicum. *Plant Biology* **2023**, *25*, 85–95, doi:10.1111/plb.13481.
40. Brito, V.L.G.; Weynans, K.; Sazima, M.; Lunau, K. Trees as Huge Flowers and Flowers as Oversized Floral Guides: The Role of Floral Color Change and Retention of Old Flowers in Tibouchina Pulchra. *Front. Plant Sci.* **2015**, *6*, doi:10.3389/fpls.2015.00362.

41. Rezende, F.M.; Clausen, M.H.; Rossi, M.; Furlan, C.M. The Regulation of Floral Colour Change in Pleroma Raddianum (DC.) Gardner. *Molecules* **2020**, *25*, 4664, doi:10.3390/molecules25204664.
42. LAMONT, B. The Significance of Flower Colour Change in Eight Co-Occurring Shrub Species. *Botanical Journal of the Linnean Society* **1985**, *90*, 145–155, doi:10.1111/j.1095-8339.1985.tb02206.x.
43. (PDF) Pigments in the Blue Pollen and Bee Pollen of Fuchsia Excorticata Available online: https://www.researchgate.net/publication/272376095_Pigments_in_the_Blue_Pollen_and_Bee_Pollen_of_Fuchsia_excorticata (accessed on 8 July 2025).
44. Delph, L.F.; Lively, C.M. THE EVOLUTION OF FLORAL COLOR CHANGE: POLLINATOR ATTRACTION VERSUS PHYSIOLOGICAL CONSTRAINTS IN FUCHSIA EXCORTICATA. *Evolution* **1989**, *43*, 1252–1262, doi:10.1111/j.1558-5646.1989.tb02572.x.
45. Teppabut, Y.; Oyama, K.; Kondo, T.; Yoshida, K. Change of Petals' Color and Chemical Components in Oenothera Flowers during Senescence. *Molecules* **2018**, *23*, 1698, doi:10.3390/molecules23071698.
46. Zhou, X.; Wang, X.; Wei, H.; Zhang, H.; Wu, Q.; Wang, L. Integrative Analysis of Transcriptome and Target Metabolites Uncovering Flavonoid Biosynthesis Regulation of Changing Petal Colors in Nymphaea 'Feitian 2.' *BMC Plant Biology* **2024**, *24*, 370, doi:10.1186/s12870-024-05078-5.
47. Smith, L.T.; Magdalena, C.; Przelomska, N.A.S.; Pérez-Escobar, O.A.; Melgar-Gómez, D.G.; Beck, S.; Negrão, R.; Mian, S.; Leitch, I.J.; Dodsworth, S.; et al. Revised Species Delimitation in the Giant Water Lily Genus Victoria (Nymphaeaceae) Confirms a New Species and Has Implications for Its Conservation. *Front. Plant Sci.* **2022**, *13*, doi:10.3389/fpls.2022.883151.
48. B.lamont, B.; Collins, B.G. Flower Colour Change in Banksia Ilicifolia: A Signal for Pollinators. *Australian Journal of Ecology* **1988**, *13*, 129–135, doi:10.1111/j.1442-9993.1988.tb00962.x.
49. Spuy, U.V. der *South African Shrubs and Trees for the Garden*; H. Keartland Publishers, 1971;
50. Liu, J.; Wang, Y.; Zhang, M.; Wang, Y.; Deng, X.; Sun, H.; Yang, D.; Xu, L.; Song, H.; Yang, M. Color Fading in Lotus (*Nelumbo Nucifera*) Petals Is Manipulated Both by Anthocyanin Biosynthesis Reduction and Active Degradation. *Plant Physiology and Biochemistry* **2022**, *179*, 100–107, doi:10.1016/j.plaphy.2022.03.021.
51. Cai, Y.-Z.; Xing, J.; Sun, M.; Zhan, Z.-Q.; Corke, H. Phenolic Antioxidants (Hydrolyzable Tannins, Flavonols, and Anthocyanins) Identified by LC-ESI-MS and MALDI-QIT-TOF MS from *Rosa Chinensis* Flowers. *J. Agric. Food Chem.* **2005**, *53*, 9940–9948, doi:10.1021/jf052137k.
52. Han, M.; Yang, C.; Zhou, J.; Zhu, J.; Meng, J.; Shen, T.; Xin, Z.; Li, H. Analysis of Flavonoids and Anthocyanin Biosynthesis-Related Genes Expression Reveals the Mechanism of Petal Color Fading of Malus Hupehensis (Rosaceae). *Braz. J. Bot* **2020**, *43*, 81–89, doi:10.1007/s40415-020-00590-y.
53. Gill, F.B.; Wolf, L.L. Foraging Strategies and Energetics of East African Sunbirds at Mistletoe Flowers. *The American Naturalist* **1975**, *109*, 491–510, doi:10.1086/283022.
54. Sohmer, S.H.; Gustafson, R. *Plants and Flowers of Hawai'i*; University of Hawaii Press, 1987; ISBN 978-0-8248-1096-2.
55. Simirgiotis, M.J.; Silva, M.; Becerra, J.; Schmeda-Hirschmann, G. Direct Characterisation of Phenolic Antioxidants in Infusions from Four Mapuche Medicinal Plants by Liquid Chromatography with Diode Array Detection (HPLC-DAD) and Electrospray Ionisation Tandem Mass Spectrometry (HPLC-ESI–MS). *Food Chemistry* **2012**, *131*, 318–327, doi:10.1016/j.foodchem.2011.07.118.
56. Lu, Y.; Wang, H.; Liu, Z.; Zhang, T.; Li, Z.; Cao, L.; Wu, S.; Liu, Y.; Yu, S.; Zhang, Q.; et al. A Naturally-Occurring Phenomenon of Flower Color Change during Flower Development in Xanthoceras Sorbifolium. *Front. Plant Sci.* **2022**, *13*, doi:10.3389/fpls.2022.1072185.
57. Guo, L.; Wang, Y.; da Silva, J.A.T.; Fan, Y.; Yu, X. Transcriptome and Chemical Analysis Reveal Putative Genes Involved in Flower Color Change in *Paeonia* 'Coral Sunset.' *Plant Physiology and Biochemistry* **2019**, *138*, 130–139, doi:10.1016/j.plaphy.2019.02.025.

58. Yoshida, K.; Miki, N.; Momonoi, K.; Kawachi, M.; Katou, K.; Okazaki, Y.; Uozumi, N.; Maeshima, M.; Kondo, T. Synchrony between Flower Opening and Petal-Color Change from Red to Blue in Morning Glory, Ipomoea Tricolor Cv. Heavenly Blue. *Proc Jpn Acad Ser B Phys Biol Sci* **2009**, *85*, 187–197, doi:10.2183/pjab.85.187.
59. Vaknin, H.; Bar-Akiva, A.; Ovadia, R.; Nissim-Levi, A.; Forer, I.; Weiss, D.; Oren-Shamir, M. Active Anthocyanin Degradation in Brunfelsia Calycina (Yesterday–Today–Tomorrow) Flowers. *Planta* **2005**, *222*, 19–26, doi:10.1007/s00425-005-1509-5.
60. Bar-Akiva, A.; Ovadia, R.; Rogachev, I.; Bar-Or, C.; Bar, E.; Freiman, Z.; Nissim-Levi, A.; Gollop, N.; Lewinsohn, E.; Aharoni, A.; et al. Metabolic Networking in Brunfelsia Calycina Petals after Flower Opening. *Journal of Experimental Botany* **2010**, *61*, 1393–1403, doi:10.1093/jxb/erq008.
61. Zipor, G.; Duarte, P.; Carqueijeiro, I.; Shahar, L.; Ovadia, R.; Teper-Bamnolker, P.; Eshel, D.; Levin, Y.; Doron-Faigenboim, A.; Sottomayor, M.; et al. In Planta Anthocyanin Degradation by a Vacuolar Class III Peroxidase in Brunfelsia Calycina Flowers. *New Phytologist* **2015**, *205*, 653–665, doi:10.1111/nph.13038.
62. Sazima, M.; Vogel, S.; Cocucci, A.; Hausner, G. The Perfume Flowers ofCyphomandra (Solanaceae): Pollination by Euglossine Bees, Bellows Mechanism, Osmophores, and Volatiles. *Pl Syst Evol* **1993**, *187*, 51–88, doi:10.1007/BF00994091.
63. Gracie, C. Pollination of Cyphomandra Endopogon Var. Endopogon (Solanaceae) by Eufriesea Spp. (Euglossini) in French Guiana. *Brittonia* **1993**, *45*, 39–46, doi:10.2307/2806859.
64. Macnish, A.J.; Jiang, C.-Z.; Negre-Zakharov, F.; Reid, M.S. Physiological and Molecular Changes during Opening and Senescence of *Nicotiana Mutabilis* Flowers. *Plant Science* **2010**, *179*, 267–272, doi:10.1016/j.plantsci.2010.05.011.
